# Carob (*Ceratonia siliqua*) as Functional Feed Is Beneficial in Yellow Mealworm (*Tenebrio molitor*) Rearing: Evidence from Growth, Antioxidant Status and Cellular Responses

**DOI:** 10.3390/antiox11091840

**Published:** 2022-09-19

**Authors:** Efthimia Antonopoulou, Nikolas Panteli, Kostantinos Feidantsis, Maria Mastoraki, Eleni I. Koutsogeorgiou, Eirini Grivaki, Theodora Papagrigoriou, Spyros P. Christias, Stavros Chatzifotis, Diamanto Lazari, Stefanos S. Andreadis, Nikos Krigas

**Affiliations:** 1 Department of Zoology, School of Biology, Aristotle University of Thessaloniki, 54124 Thessaloniki, Greece; 2 Institute of Marine Biology, Biotechnology and Aquaculture, Hellenic Centre for Marine Research, Gournes Pediados, 71003 Heraklion, Greece; 3 Institute of Plant Breeding and Genetic Resources, Hellenic Agricultural Organization—Dimitra, 57001 Thermi, Greece; 4 Laboratory of Pharmacognosy, Faculty of Pharmacy, School of Health Sciences, Aristotle University of Thessaloniki, 54124 Thessaloniki, Greece; 5 Ypergaros Limited Liability Company, Megala Kalyvia, 42100 Trikala, Greece

**Keywords:** insects, alternative feeds, lipid peroxidation, antioxidant enzymes, apoptosis, phenolic content, Fabaceae

## Abstract

In terms of sustainability and circular economy, agricultural by-products may be efficiently reused in insects’ rearing for high-quality protein sources in human diet and animal feeds. The present study aimed to explore whether the utilization of carob pods as feeding substrate may beneficially affect *Tenebrio molitor*’s growth, nutritional value, antioxidant status and cellular responses. Increasing levels of milled whole carob pods (0, 25, 50, 75, 100%) were used as alternative wheat bran (control) substrates for yellow mealworm rearing, while growth performance, proximate composition, total phenolic content, antioxidant enzyme activity and the expression of stress- and apoptotic-related proteins were evaluated in larvae. The results showed that carob pods’ content up to 75% did not significantly differentiate larvae weight, development time and total dry matter. Larvae total phenolic content and antioxidant activity exhibited a significant increase at 75% content. Although the antioxidant enzymes’ activity decreased at both 25 and 50% levels, higher carob content levels (75 and 100%) resulted in no significant changes compared to the control. Carob pods led to decreased apoptotic indicators and the low expression of most stress-related proteins compared to the control. The present findings demonstrate that carob pods and their antioxidant properties exert beneficial effects on *T. molitor*’s rearing and nutritional status, although 100% carob content may impact adversely the larvae due to the high amounts of carob tannins.

## 1. Introduction

In a period when hunger affects nearly 10% of the world’s population [1], the development of alternative protein sources is urgently needed to alleviate global poverty [2]. Insects are gradually emerging as a potential high-quality and sustainable component of both animal and human diets [3]. Organizations such as the Food and Agriculture Organization of the United Nations (FAO) advocate, especially in Western societies, the nutritional, economic and environmental advantages of insects’ commercialization as a novel food and feed [4,5]. Although their nutritional value depends on several factors including species and development stage [6], several edible insects tend to be an efficient source of protein, energy, monounsaturated and/or polyunsaturated fatty acids, and micronutrients such as copper, iron and magnesium, with a well-balanced amino acid profile that meets human requirements [4,7]. Furthermore, insects’ rearing for food and feed could mitigate environmental degradation due to lower greenhouse gas emissions and limited requirements in breeding space and resources compared to livestock production [8,9]. Simultaneously, insects’ mass production can fit well with the circular economy model, which embraces the continual and efficient recycling and reuse of materials, by-products or wastes as resources in an effort to minimize waste generation [10]. Insects’ production may exploit several low-value organic, industrial and agricultural by-products as rearing substrates which insects can efficiently be recycled and bioconverted into high-value protein [8,11,12]. In particular, recent studies demonstrated that by-product substrates may increase the final weight and enhance the antioxidant capacity of insects [11,13].

The inclusion of insect larvae meals in the diets of teleosts have been extensively studied with very promising results. In European sea bass (*Dicentrarchus labrax*) and gilthead sea bream (*Sparus aurata*), the dietary incorporation of insect larvae meals has no adverse effects on growth performance and feed utilization [14,15], while simultaneously enhancing the abundance of beneficial bacteria in their gut [16]. However, extensive insect meal inclusion may cause imbalance in the gut microbiota [17] and concomitant stress may induce autophagy in the liver of sea bream [18]. The inclusion of medicinal and aromatic plants in the rearing of *Tenebrio molitor* larvae has been shown to benefit their growth performance, resulting in higher survival and total larval weight, and improvement of the fatty acid profile of the larvae [11]. In addition, the substrate enrichment with phenolics from medicinal and aromatic plants may not only enhance the growth performance but can also increase the antioxidant capacity of the larvae [11].

Carob or locust bean (*Ceratonia siliqua* L.) is an evergreen tree of the Fabaceae family, which due to its drought- and salt-tolerant nature is widely cultivated around the Mediterranean basin for its sugary pods and gum-containing seeds [19,20]. Carob pods and seeds are used as raw materials in the production of food, cosmetics and medications [21,22]. In terms of nutritional value, carob pods contain high levels of carbohydrates (mainly sucrose, glucose, fructose and maltose) and adequate amounts of crude fibers (cellulose and hemicellulose), associated with low protein (1–5%) and fat content [21,23,24]. Additionally, carob serves as a good source of several minerals such as phosphorus, potassium and iron and is rich in vitamins such as E, D and C [24,25]. Furthermore, several phenolic compounds including pyrogallol, catechin, gallic acid, chlorogenic and protocatechuic act as antioxidants are present in carob pods [26]. Therefore, carob pods have been reported to enhance the antioxidant enzymes activity, exerting beneficial effects on oxidative stress and lipid peroxidation in animals [27,28].

Considering that carob trees naturally occur as wild-growing plants around the Mediterranean region and given that high quantities of carob pods are usually not harvested from existing (or abandoned) cultivations or their industrial by-products are discarded without efficient utilization, the present study aimed to explore and evaluate the effects of milled carob pods as a novel feeding substrate of the yellow mealworm, *Tenebrio molitor* L. (Coleoptera: Tenebrionidae). The latter is the first insect species authorized by the European Commission (Implementing Regulation EU 2021/882) for novel food and feed consumption in the European market. In specific, yellow mealworms were reared in substrates containing different inclusion levels of milled carob pods (0, 25, 50, 75 and 100%) as wheat bran substitutes, and the effect on insects’ growth, antioxidant status and cellular responses was examined. To the authors’ knowledge, this is the first report that reports on the utilization of carob pods as alternative substrate for insects in general.

## 2. Materials and Methods

### 2.1. Substrate Preparation

**Figure 1:**
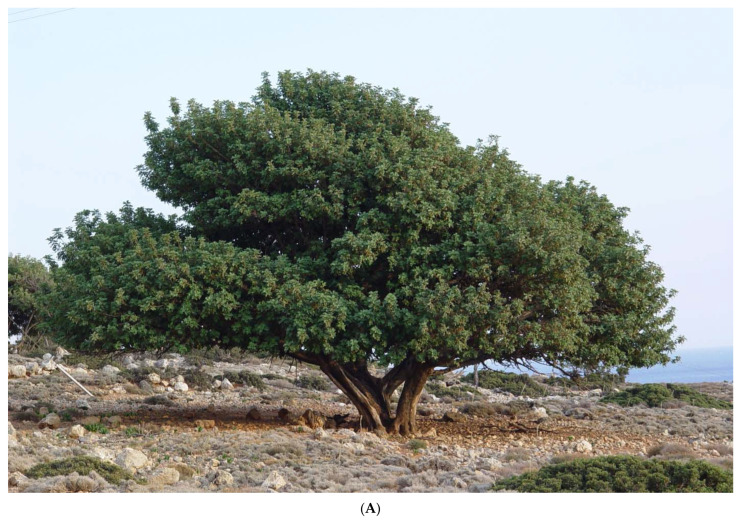

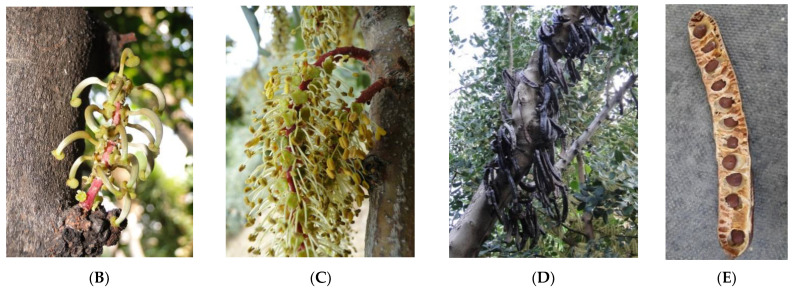
Hermphroditic carob tree in Crete, Greece (**A**) with female (**B**) and male (**C**) inflorescences, and carob pods (**D**) with mature seeds (**E**).

Dried carob pods were hand-collected from cultivated carob trees (Figure 1) at the end of August 2021 (Keratokampos, Crete, Greece).

**Figure 2:**
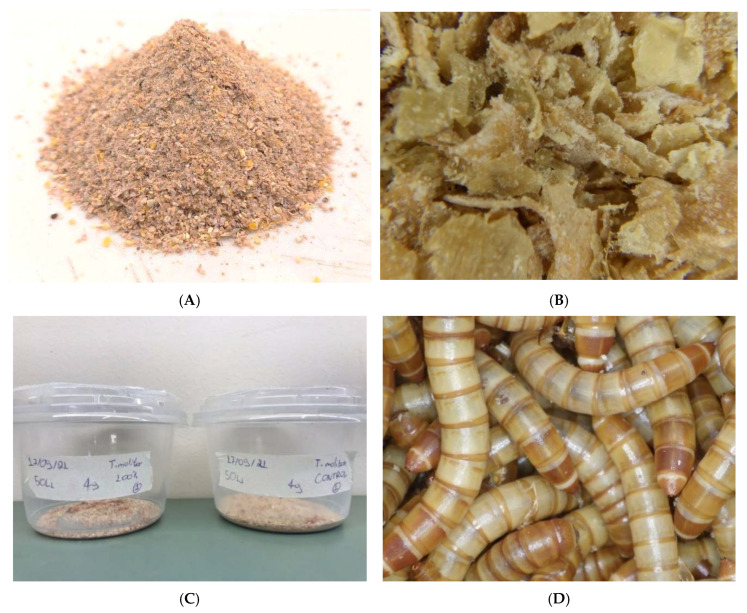
Representative illustration of milled whole carob pods (**A**) and substrate with 50% milled carob (**B**) used for rearing (**C**) in transparent plastic cylindrical vials (7.5 cm in diameter, 8.8 cm in height) containing *Tebebrio molitor* larvae (**D**).

The milled whole carobs’ insect feed (Figure 2A) was produced at the facilities of the company “YPERAGROS” using a specialized feed production machinery (A. P. Klimatsidas & SIA OE, Larisa, Greece) with an electronic weighing scale of up to 1 t (capacity vertical hopper 2000 L), four load cells, a silo (dimensions 1 m × 2 m × 1.5 m), a pneumatic hammer mill of 15 HP with steel knives for processing materials (production potential of 1 t/h), as well as a quantity regulator at the input, a set of three sieves, Φ130 auger with a sieve from the hammer mill to the 1 t mixer of a 4 HP motor with a pulley, belts, a hopper for receiving the by-products, a manhole and control glass. Prior to milling the whole carob pods, all necessary actions have been taken for the cleaning of the machinery to prevent mixture with other raw materials.

A mixture of the milled carob pods and wheat bran purchased from a local store was prepared and this was used as a substrate in the following percentages: 0, 25, 50 (Figure 2B), 75 and 100%. A substrate consisting of pure wheat bran (100%) was used as control.

### 2.2. Pilot Insect Rearing

*Tenebrio molitor* used in this study were derived from a stock colony that was maintained at the Entomology Lab of the Institute of Plant Breeding and Genetic Resources (Thermi, Greece) since 2019. The insects were kept under standard conditions at 26 °C and 60% relative humidity under an 8:16 h (L:D) photoperiod, and they were fed with wheat bran following published protocols [11]. Fresh carrot slices were provided to the insects three times per week as a moisture source. Newly-hatched first instar larvae (<48 h) were used for the experimentation.

### 2.3. Experimental Design

To acquire newly-hatched first instar larvae, adults were placed in white plastic trays (20 × 11 × 4 cm) and left to oviposit for seven days in a small quantity of wheat bran (5 g). Afterwards, the adults were removed and eggs stuck on the bottom of the tray were monitored daily. Newly-hatched first instar larvae of *T. molitor* (<48 h-old) were collected every other day with a size one paint brush (Artist’s LoftTM, MSPCI, TX, USA). These were weighed in groups of 50 using a high precision scale (KERN, ALJ 250-4AΜ) and were placed in five separate transparent plastic cylindrical vials (7.5 cm in diameter, 8.8 cm in height; Figure 2C). Each vial contained a different substrate (four treatments and control). A total of six replicates was used for each treatment, resulting in a total of 300 first instar larvae in the treated and control groups, respectively. Moisture was provided to the larvae through 0.3 ± 0.05 g fresh carrot slices twice a week, while old pieces were removed from the vials. Larvae were left to feed undisturbed for a period of four weeks, after which the larvae were counted and weighed as a group. After the four-week interval, larvae were monitored biweekly in the same fashion, while the plastic vials were checked during the week in case more feed was necessary (Figure 2D). This process continued until the first pupa emerged into each cup, upon which the final instar larvae were placed into separate plastic zip lock bags depending on the treatment and substrate. The development and survival data (weight, number of larvae and time of the first appearance of a pupa) were imported and processed in Microsoft Excel 2019 (v16.0).

### 2.4. Insect Rearing for Chemical Analyses

For each experimental treatment, three replicates of approximately 1,000 adults were collected with featherweight forceps (BioQuip Products, Rancho Dominguez, CA, USA) and were placed in plastic insect breeding boxes (60 × 40 × 14.5 cm) (Beekenkamp Verpakkinken BV, Maasdijk, The Netherlands) with 1.0 kg of mixture of milled carob pods and wheat bran as food source and oviposition substrate. After one week, adults were removed, whereas glued eggs on the bottom of the boxes and newly hatched larvae remained in the plastic boxes for a period of approximately three months. A fresh substrate of each mixture was added every two weeks, while fresh carrot slices were supplied twice a week until the occurrence of the first pupa. Last instar larvae were collected and were stored under a vacuum at −20 °C for further analyses.

### 2.5. Proximate Composition

The proximate composition of the substrates and the insect larvae were determined according to AOAC [29]. To facilitate the insect larvae analyses and to prevent spoilage, all proximate composition analyses were performed using freeze-dried (Telstar Cryodos, Terrassa, Spain) and finely chopped insect larvae, except for the total dry matter, which was measured before the freeze-drying step. The dry matter content was assessed by dehydrating it at 90 °C until a constant weight, and the ash content was determined after incineration at 700 °C for 7 h. Fat content was determined with chloroform–methanol extraction [30], and protein content was measured according to the Dumas method using a nitrogen analyzer (FP-528, Leco corporation, St. Joseph, MI, USA). A nitrogen-to-protein conversion factor of Kp = 6.25 was used for the substrates and larvae, and an additional nitrogen-to-protein conversion factor of Kp = 4.76 was used for the insect larvae following Janssen et al. [31] to facilitate the comparison with other studies. Energy content was determined with a bob calorimeter (6300, Parr Instrument Company, St. Moline, IL, USA).

To determine the chitin content of the larvae, three analyses were performed. Initially, the acid detergent fiber (ADF) fraction of the insects was measured by boiling the insect sample in a solution of cetyltrimethylammonium bromide/H_2_SO_4_ (0.5 mol/L) using Fibretherm (C. Gerhardt GmbH & Co., Königswinter, Germany) and the subsequent drying of the filtration residue. Due to the complex structure of the insect cuticle, chitin is closely linked to cuticular proteins and minerals [32]. The mineral content of ADF was determined by incineration of the ADF residue at 700 °C for at least four h. The protein content of ADF was determined by measuring the nitrogen content of the filtration residue according to Goering & Van Soest [33] using a nitrogen analyzer and multiplying the nitrogen content by 6.25. Chitin was determined according to Marono et al. [34] using the formula: Chitin% = ADF% − (ash% of ADF × ADF%/100) − (protein% of ADF × ADF%/100).

### 2.6. Determination of Total Phenolic Content and Antioxidant Activity

#### 2.6.1. Preparation of Extracts

For the preparation of the *T. molitor* larvae extract, the protocol of Andreadis et al. [11] was followed, with some modifications. More specifically, 220 mg of deep-frozen larvae (at −80 °C) was homogenized in 5 mL of MeOH_(aq)_ 80% for 5 min using an Ultra-Turrax T25 dispersing instrument (IKA Labortechnik). The extraction was proceeded overnight at 4 °C, and the next day the samples were centrifuged (3.200 rpm, 20 min). The supernatants were collected and dried in vacuo (40 °C, IKA RV 3 eco, rotary evaporator).

The protocol of Frühbauerová et al. [35] was followed for the preparation of the substrates’ extracts, with some modifications: 200 mg of dried, milled carob substrate was extracted in 5 mL of aqueous methanol 80%, acidified with 0.3% formic acid, using an ultrasonic bath (Cole-Parmer 8893) for 10 min. The extracts were subsequently filtered, and the precipitate was re-extracted for a second time. The filtrates were combined and evaporated in vacuo (40 °C, IKA RV 3 eco rotary evaporator).

#### 2.6.2. Determination of Total Phenolic Content (TPC)

The dried extracts were re-dissolved in DMSO at a concentration of 10 mg mL^−1^ and their total phenolic content was determined according to the Folin–Ciocalteu method [36] as follows: 20 μL of each sample was added to a test tube, along with 2500 μL of deionized water and 400 μL of Folin–Ciocalteu reagent (F9252, Sigma-Aldrich, Burlington, MA, USA). The test tubes were left to stand in the dark for 8 min, at room temperature. Subsequently, 500 μL of Na_2_CO_3_ 7% was added to the reaction mixture, followed by incubation at 40 °C for 30 min in the dark. The samples were afterwards left to cool until they reached room temperature and their absorbance was measured at λ = 750 nm, using a UV-vis spectrophotometer (UV-1700 PharmaSpec, Shimadzu, Kyoto, Japan). The total phenolic content was calculated by means of a gallic acid standard curve (0–1.5 mg mL^−1^, R^2^ = 0.947) and was expressed as micrograms of gallic acid equivalents per gram of frozen weight or dry weight (μg GAE g^−1^ FW or μg GAE g^−1^ DW) in the case of the *T. molitor* larvae extracts or the carob substrate extracts, respectively. All assays were performed in triplicate.

#### 2.6.3. Determination of Antioxidant Activity

A modified protocol of Risaliti et al. [37] was followed for the determination of the extracts’ antioxidant (radical scavenging) activity. Briefly, 20 μL of sample was incubated with DPPH (D211400, Sigma-Aldrich) (0.1 mM in MeOH) in the dark for 30 min, at room temperature, and the absorption of the reaction mixture was read at 517 nm using a UV-vis spectrophotometer (UV-1700 PharmaSpec, Shimadzu, Kyoto, Japan). The extracts’ radical scavenging activity was calculated by means of a Trolox standard curve (0–500 μmol L^−1^, R^2^ = 0.992). The results were expressed as nanomoles of Trolox equivalents per gram of frozen weight or dry weight (nmol TE g^−1^ FW or nmol TE g^−1^ DW) for the *T. molitor* larvae extracts or the carob substrate extracts, respectively. All assays were performed in triplicate.

### 2.7. Assays of Antioxidant Enzymes and Lipid Peroxidation

Frozen larvae were prepared for the measurement of antioxidant enzyme activity according to the protocol described in Salach [38]. Specifically, the larvae were immediately homogenized in ice-cold phosphate buffer (50 mM, pH 7.4) 10% (*w*/*v*) using Omni international homogenizer (USA) at 22,000 rpm for 20 s, each with 10 s intervals. The homogenate was centrifuged at 2000× *g* in a cooling centrifuge at 4 °C for 15 min and the supernatant was stored. The supernatant was freeze-thawed three times to completely disrupt the mitochondria. Then, the supernatant was again centrifuged at 6000× *g* in a cooling centrifuge at 4 °C for 15 min, and the yielded supernatant that contained the cytosolic and mitochondrial enzymes was stored for enzyme assays and the determination of lipid peroxidation.

The terminal product, malondialdehyde (MDA), which formed in the decomposition of polyunsaturated fatty acids mediated by free radicals, was quantified as levels of thiobarbituric acid reactive substances according to the method of Buege and Aust [39].

The enzymatic activities (Vmax) were determined spectrophotometrically at 18 °C and all assays were based on well-established protocols [40,41,42].

Total superoxide dismutase (mitochondrial Mn- and cytosolic Cu/Zn-superoxide dismutase, SOD EC 1.15.1.1) activity was assayed by monitoring NADH oxidation. SOD activity was assayed by assessing the inhibition of NADH oxidation using β-mercaptoethanol in the presence of EDTA and Mn as a substrate. NADH solution was prepared daily. The assays were run by adding 0.80 mL of 50 mM phosphate buffer (pH 7.4), 55 μL EDTA/Mn solution of 100/50 mM, 40 μL NADH solution of 7.5 mM, and different volumes of larvae extract to the cuvette sequentially. The reaction was then initiated by adding 100 μL of 10 mM β-mercaptoethanol solution. The changes in the absorbance of NADH at 340 nM per min was followed (ε = 6.22 mM^−1^ cm^−1^ at 340 nm). One unit of SOD activity is defined as the amount of larvae extract required to inhibit the rate of NADH oxidation of the control by 50%, while the specific activity is expressed as units per mg of protein.

Catalase (CAT, EC 1.11.1.6) activity was assayed as follows: 1 mL of 50 mM phosphate buffer (pH 7.4) and 10 μL of larvae extract was added to the cuvette. The reaction was then initiated by the addition of 300 μL of 30 mM H_2_O_2_, prepared by diluting 0.34 mL of 30% H_2_O_2_ to 100 mL of 50 mM phosphate buffer (pH 7.4). Catalase activity was determined following the changes in the absorbance of H_2_O_2_ at 240 nm (ε = 0.0394 mM^−1^ cm^−1^ at 240 nm) and is expressed as micromoles per minute per milligram of protein.

Glutathione reductase (GR, EC 1.8.1.7) activity was assayed as follows: 1 mL of 50 mM sodium phosphate (pH 7.4) containing 2 mM EDTA and 0.15 mM NADPH and 10 μL of larvae extract were added to the cuvette. The reaction was then initiated by the addition of 10 μL of 1 mM GSSG. GR activities were determined following the changes in the absorbance of NADPH per min at 340 nm (ε = 6.22 mM^−1^ cm^−1^).

Enzymatic activities were expressed as units per milligram of protein or micromoles per minute per milligram of protein.

### 2.8. Preparation for Immunoblotting

The preparation of samples for SDS-PAGE, the quantification of caspases and ubiquitinated proteins and immunoblot analysis were based on well-established protocols. Specifically, frozen larvae were immediately homogenized in 3 mL g^−1^ of cold lysis buffer (20 mM β-glycerophosphate, 50 mM NaF, 2 mM EDTA, 20 mM Hepes, 0.2 mM Na3VO4, 10 mM benzamidine, pH 7, 200 μM leupeptin, 10 μΜ trans-epoxy succinyl-Lleucylamido-(4-guanidino) butane, 5 mM dithiotheitol, 300 μΜ phenyl methyl–sulfonyl fluoride (PMSF), 50 μg mL^−1^ pepstatin, 1% *v*/*v* Triton X-100), and were extracted on ice for 30 min. The samples were centrifuged (10,000× *g*, 10 min, 4 °C) and the supernatant was boiled with 0.33 volumes of SDS/PAGE sample buffer (330 mM Tris-HCl, 13% *v*/*v* glycerol, 133 mM DTT, 10% *w*/*v* SDS, 0.2% *w*/*v* bromophenol blue). Protein concentration was determined by using the BioRad protein assay.

For the SDS-PAGE, equivalent amounts of proteins (50 μg), from the larvae of each experimental treatment, were separated either on 10% and 0.275% (*w*/*v*) acrylamide and bisacrylamide, respectively, followed by electrophoretic transfer onto nitrocellulose membranes (0.45 μm, Schleicher & Schuell, Keene, NH 03431, USA).

The resulting nitrocellulose membranes were subjected to overnight incubation with: polyclonal rabbit anti-bcl2 (7973, Abcam), polyclonal rabbit anti-bax (B-9) (7480, Santa Cruz Biotechnology, Dallas, TX, USA), monoclonal mouse anti-HSP70 (H5147, Sigma), monoclonal mouse anti-HSP90 (H1775, Sigma), anti-HSP60 (12165, Cell Signaling, Beverly, MA, USA), polyclonal rabbit anti-phospho-p38 MAP kinase (9211, Cell Signaling, Beverly, MA, USA), and polyclonal rabbit anti-p38 MAPK (9212, Cell Signaling, Beverly, MA, USA). Quality transfer and a protein-loading Western blot were assured by the Ponceau stain and actin (anti-β actin 3700, Cell Signaling, Beverly, MA, USA). The bands were detected by enhanced chemiluminescence, while quantification was applied through laser-scanning densitometry (GelPro Analyzer Software, GraphPad, San Diego, CA, USA).

### 2.9. Statistics

Changes in biochemical responses (mean ± SD) were tested for significance at the 5% level by using a one-way analysis of variance (ANOVA) (GraphPad Instat 3.0). Post-hoc comparisons were performed using the Dunnett’s test (for comparisons with 0%—control) and Tukey’s test (for comparisons between all examined carob contents). Values are presented as means ± S.D. Correlations were performed using the non-parametric Spearman’s correlation analysis.

## 3. Results

### 3.1. Substrates’ Proximate Composition

The proximate composition of the five experimental substrates is presented in Table 1. Dry matter was similar among the substrates and ranged from 88.6 to 90.2%. Crude protein was higher in the 50% substrate (17.2 ± 0.5%) followed by the 0% (16.6 ± 0.2%), and was lower in the 100% substrate (14.2 ± 1.9%). Fat and energy contents correlated negatively with the milled carob pod content levels (r = −0.884 and r = −0.927, respectively, *p* < 0.001). The greatest differences among the substrates were observed in the ash and fat contents. The ash content ranged from 5.1% in the 25% carob substrate to 9% in the 100% carob substrate and correlated positively with the carob content levels (r = 0.873, *p* < 0.001).

### 3.2. Growth Performance

#### 3.2.1. Individual Larval Weight

**Figure 3:**
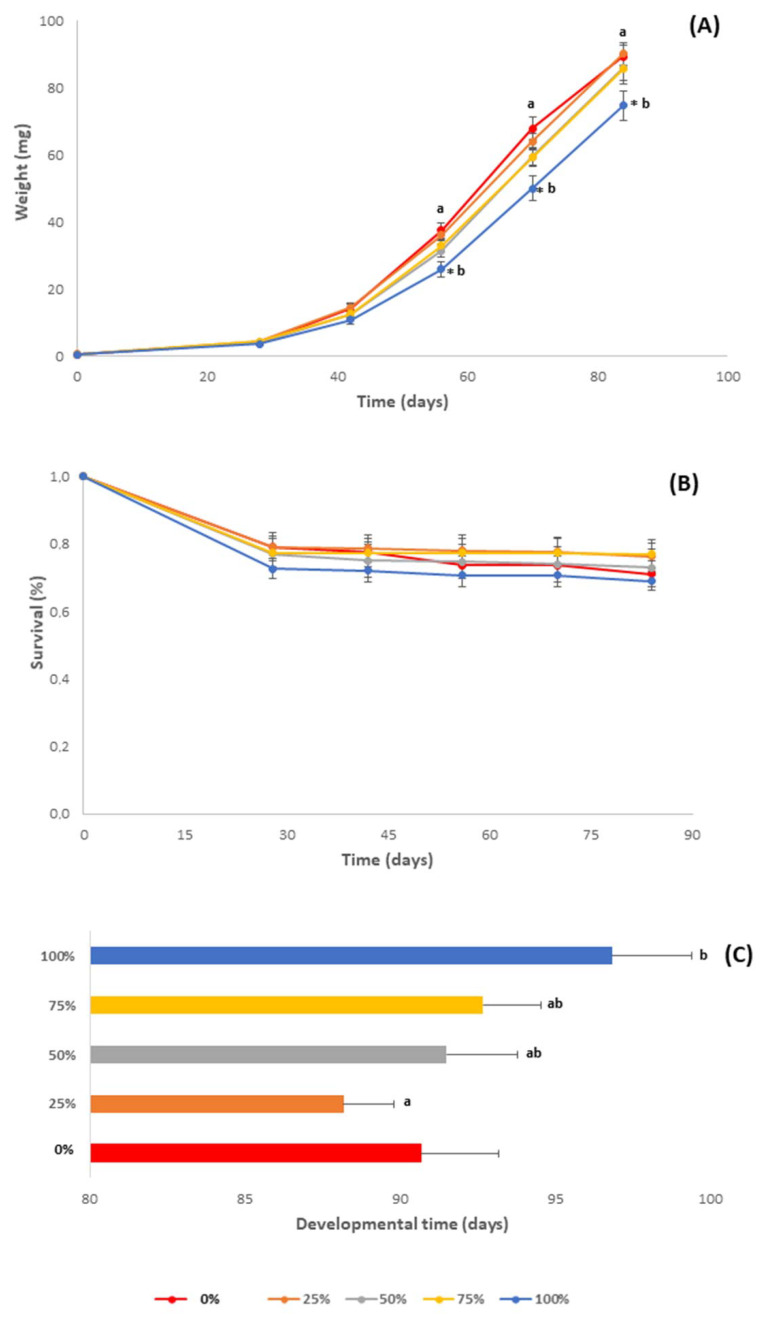
(**A**) Average larval weight (mg), (**B**) survival rate (%) and (**C**) development time (days) of *Tenebrio molitor* larvae reared on substrates containing different content levels of milled carob pods (0, 25, 50, 75 and 100%). In all cases, values represent means ± SE (*n* = 6). Asterisk (*) indicates *p* < 0.05 compared to 0%, while lower case letters indicate *p* < 0.05 compared to different carob contents (%).

Variations in larval growth were recorded and expressed as individual larval weight (Figure 3A), which at the end of the bioassay fluctuated between 74.6 mg (100% carob) and 90.3 mg (25% carob and wheat bran mixture). According to our results, the larvae of *T. molitor* fed with 100% milled carob pods displayed significantly lower individual larval weight at the end of the bioassay compared to all other treatments, including 0% (F = 4.079, df = 4, *p* < 0.05). On the other hand, no significant difference was observed between the individual larval weight of larvae reared on 0, 25, 50 and 75% substrates. A correlational analysis revealed that the final larval weight correlated positively with the fat and energy content of the substrates (r = 1, *p* = 0.017 for both nutrients).

#### 3.2.2. Survival Rate

The survival rate of *T. molitor* larvae overtime is shown in Figure 3B. Larvae of *T. molitor* fed the 25 and 75% substrates at the end of the bioassay displayed higher survival rates (76 and 77%, respectively) than those fed the 50, 100 and 0% milled carob pods (73, 69 and 71%, respectively) (F = 0.561, df = 4, *p* = 0.693). A lower survival rate among all treatments was observed in larvae fed with 100% milled carob pods. However, in all time intervals, differences in the survival rates among the different dietary treatments were not found to be significant (F_4 weeks_ = 0.366, df = 4, *p* = 0.831; F_6 weeks_ = 0.387, df = 4, *p* = 0.816; F_8 weeks_ = 0.440, df = 4, *p* = 0.779; F_10 weeks_ = 0.419, df = 4, *p* = 0.793).

#### 3.2.3. Development Time

Regarding the development time (Figure 3C), no significant difference was found among treatments (F = 1.747, df = 4, *p* = 0.172), although larvae in the 25% treatment showed a lower development time compared to all other treatments (88.2 days), including 0% (90.7 days). Comparatively, the larvae fed the 100% milled carob pods displayed the longest development time (96.8 days). A correlational analysis revealed that the development time correlated negatively with the fat and energy content of the substrates (r = −1, *p* = 0.017 for both nutrients).

### 3.3. Proximate Composition of the T. molitor Larvae

The proximate composition of the *T. molitor* larvae fed the different substrates is presented in Table 2. Total dry matter and ash contents were not affected by the carob content. However, the ash content correlated negatively with the level of carob meal content (r = −0.622, *p* < 0.05). The protein content was higher in TM larvae fed the substrates with 75% or 100% carob meal (49.1–49.4%) compared to the 0% (44.8 ± 0.6%), and a strong significant positive correlation was observed between larvae protein content and carob meal incorporation (r = 0.851, *p* < 0.001). The fat content was significantly higher in TM larvae fed 0% carob (23.8 ± 0.4%) compared to the 50% group (20.7 ± 1.1%), while the rest of the groups exhibited similar fat contents among each other. Energy content correlated negatively with the carob meal content level (r = −0.709, *p* < 0.01) and was significantly higher in TM larvae fed 0% and 25% (25.1–25.3 MJ/Kg) carob compared to the 75% and 100% groups (24.2 MJ/Kg). Chitin content was significantly lower in the 0 and 25% groups (6.4–7.1%) compared to the other groups (8.2–8.4%) and showed a positive correlation with the carob content (r = 0.709, *p* < 0.01). The fat and energy content correlated positively with the final larval weight (r = 0.513 and 0.589, respectively, *p* < 0.05) and the protein and chitin content correlated negatively (r = −0.72 and −0.676, respectively, *p* < 0.01). 

### 3.4. Total Phenolic Content of C. siliqua Substrates and T. molitor Larvae Extracts

**Figure 4:**
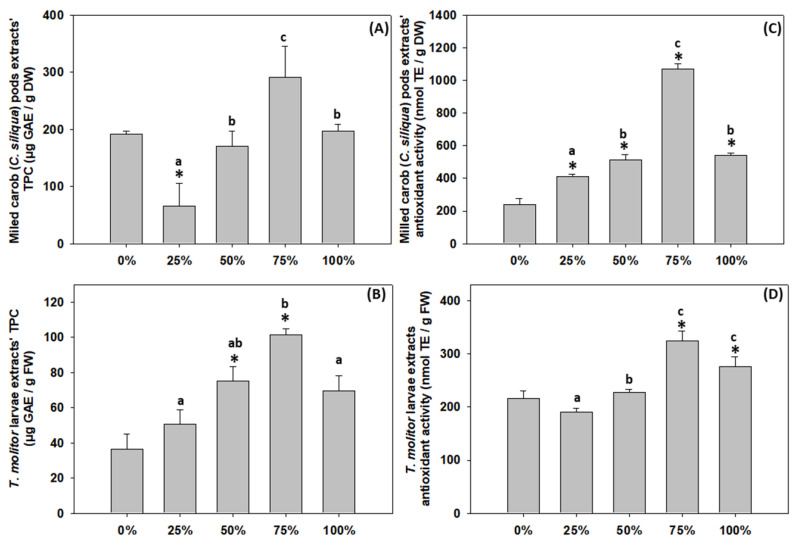
Total phenolic contents (**A**,**B**) and antioxidant activities (**C**,**D**) of carob pods used as feeding substrate at different content levels (0, 25, 50, 75, 100%) and *Tenebrio molitor* larvae, respectively. Values represent means ± SD. Asterisk (*) indicates *p* < 0.05 compared to 0%, while lower case letters indicate *p* < 0.05 compared to different carob contents (%).

Regarding the feeding substrates (Figure 4A), the 25% content of milled carob pods resulted in a significant reduction of the extracts’ total phenolic content, while no changes were observed in the substrates of 50, 75 and 100% content compared to the control group. However, 75% carob content was statistically higher compared to 25%.

As demonstrated in Figure 4B, the total phenolic content of the *T. molitor* extracts increased in all cases but displayed a statistically significant increase in larvae fed the 50 and 75% substrates. The highest total phenolic content was found in the 75% carob content group, and it was statistically significant compared to the 25 and 100% groups.

### 3.5. Antioxidant Activity of C. siliqua Substrates and T. molitor Larvae Extracts

Figure 4C depicts that in the case of the substrate extracts, there was a gradual increase in radical scavenging activity (RSA) in response to the increase in the percentage (%) of carob content. However, a significant elevation compared to the 0% group was apparent in the 50, 75 and 100% substrates. In contrast, the 100% content of carob pods resulted in a significant decrease compared to the 75% group.

Interestingly, the extract from *T. molitor* larvae that had been fed with 75% carob pods content showed the highest RSA, while all other substrates resulted to no statistically significant changes compared to the 0% group (Figure 4D).

### 3.6. Lipid Peroxidation and Antioxidant Defence of T. molitor Larvae

**Figure 5:**
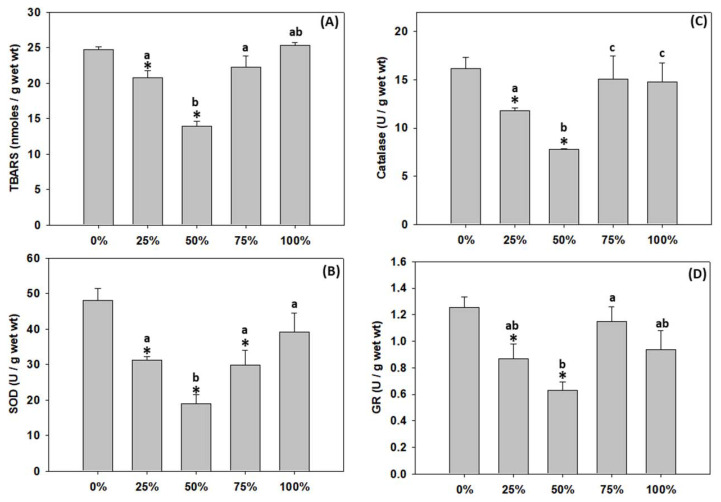
(**A**) TBARS levels, (**B**) SOD, (**C**) catalase and (**D**) GR enzymatic activity levels (mean ± SD) of *Tenebrio molitor* larvae reared on substrates containing different content levels of milled carob pods (0, 25, 50, 75 and 100%). Asterisk (*) indicates *p* < 0.05 compared to 0%, while lower case letters indicate *p* < 0.05 compared to different carob contents (%).

Milled carob pods at 100% content level had no effect on TBARS levels (Figure 5A) nor any of the antioxidant enzymes’ activity (Figure 5B–D) compared to 0%. The 75% content revealed a similar pattern, with the only difference being that SOD activity statistically decreased compared to 0% (Figure 5B). Increasing carob up to 50% content resulted in parallelly decreasing levels of both TBARS (Figure 5A) and all enzymes’ activities (Figure 5B–D). Interestingly, 50% carob content presented the lowest levels regarding all the above-mentioned parameters (Figure 5A–D).

### 3.7. Heat Shock Protein Response (HSP) of T. molitor Larvae

**Figure 6:**
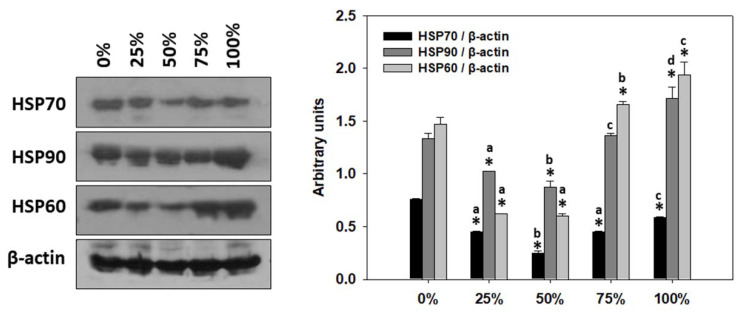
HSP70, HSP90 and HSP60 induction levels (mean ± SD) of *Tenebrio molitor* larvae reared on substrates containing different content levels of milled carob pods (0, 25, 50, 75 and 100%). Asterisk (*) indicates *p* < 0.05 compared to 0%, while lower case letters indicate *p* < 0.05 compared to different carob contents (%).

Figure 6 (complete original immunoblocks in Supplementary Material Figures S1 and S4) depicts the detected changes in HSPs’ induction levels of *T. molitor* larvae under the effect of milled carob content in the substrate (%). Regarding HSP70, all content levels revealed decreased trends compared to 0%. The lowest levels, however, were observed at 50%. HSP90 levels similarly decreased at 25 and 50% carob content compared to 0%, while 75% depicted no statistical differences compared to 0%. On the other hand, the 100% carob content resulted in significantly higher HSP90 levels than that of 0%. A similar pattern was observed regarding HSP60 induction levels, with the only difference that both 75 and 100% carob content resulted in significantly higher levels compared to that of 0%.

### 3.8. Response of T. molitor larvae p38 MAPK

**Figure 7:**
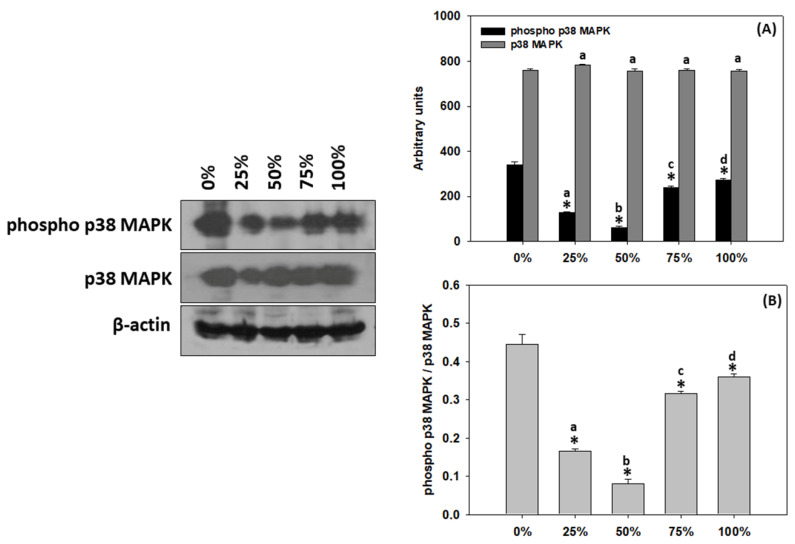
(**A**) Phosphorylated p38 MAPK, p38 MAPK and (**B**) phosphorylated p38 MAPK/p38 MAPK levels (mean ± SD) of *Tenebrio molitor* larvae reared on substrates containing different content levels of milled carob pods (0, 25, 50, 75 and 100%). Asterisk (*) indicates *p* < 0.05 compared to 0%, while lower case letters indicate *p* < 0.05 compared to different carob contents (%).

As depicted in Figure 7A, the different content levels of milled carob pods resulted in no differences regarding the p38 MAPK levels of *T. molitor* larvae (see complete original immunoblocks in Supplementary Material Figures S2 and S4). However, the activation of p38 MAPK (Figure 7A,B) changed significantly due to the carob content. Specifically, all content levels revealed decreased trends compared to 0% carob content, with the 50% carob content displaying the lowest phosphorylation levels, while the 75 and 100% group were statistically higher compared to the 25 and 50% ones.

### 3.9. Apoptosis in T. molitor Larvae

**Figure 8:**
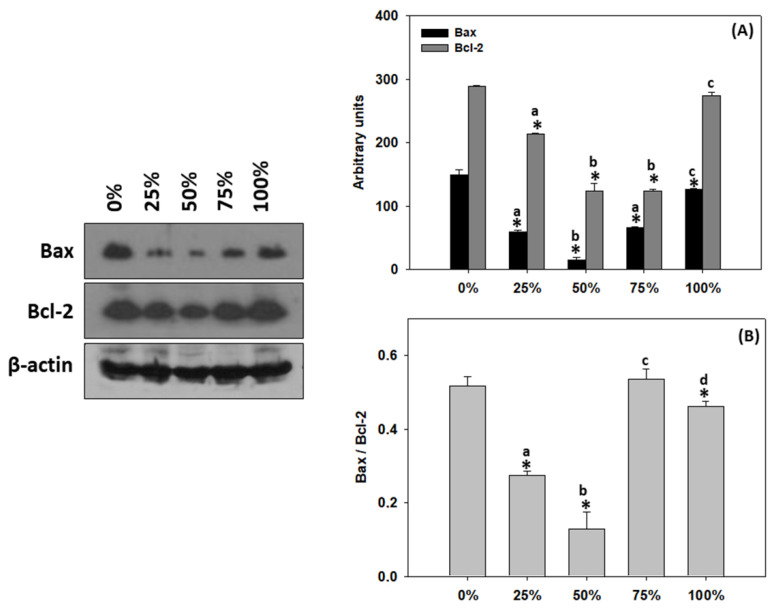
(**A**) Bax, Bcl-2 and (**B**) Bax/Bcl-2 levels (mean ± SD) of *Tenebrio molitor* larvae reared on substrates containing different content levels of milled carob pods (0, 25, 50, 75 and 100%). Asterisk (*) indicates *p* < 0.05 compared to 0%, while lower case letters indicate *p* < 0.05 compared to different carob contents (%).

Milled carob pods content levels resulted in changes—mostly decreases—in both Bax and Bcl-2 levels. Specifically, 25 and 75% carob content resulted in decreased Bax levels compared to 0%, while 50% revealed the most significant trend. Furthermore, 100% carob content, although statistically higher compared to 25%, 50% and 75%, remained statistically decreased compared to 0%. The 100% carob content resulted in no Bcl-2 changes compared to 0%. However, 25% carob content resulted in statistically decreased levels compared to 25%, while larvae raised on bran of 50% and 75% carob content showcased the lowest Bcl-2 levels compared to all content levels (Figure 8A, (complete original immunoblots in Supplementary Materials Figures S3 and S4). Bax/Bcl-2 ratio levels decreased at all content levels, except for 75%, which presented no statistical differences compared to 0%. Increasing the carob content resulted in parallel decreasing Bax/Bcl-2 levels with 50% presenting the lowest levels, while 100% carob content resulted in significantly higher ratio levels compared to 25% and 50% (Figure 8B).

## 4. Discussion

To the authors’ best knowledge, this is the first report regarding the utilization of carob pods as alternative novel substrate for edible insect rearing such as *T. molitor*. To date, carob inclusion in animal feed has been mostly employed in livestock but not insects. The larvae of *T. molitor* fed with the 25% carob mixture displayed higher survival rates and lower development time than all other substrates, including the 0% (wheat bran). This evidence renders milled whole carob pods a promising substrate for the development of *T. molitor*; however further studies are required to validate these results. Interestingly enough, larvae fed with 100% milled carob pods displayed the lowest individual larval weight but contained the highest amount of protein among treatments. The latter could be attributed to the fact that the increased addition of carob pods may worsen the above-measured parameters, as has been previously observed in growing rabbits [28]. Moreover, it should be highlighted that excessive dietary carob inclusion may adversely affect feed intake and growth due to the high level of condensed tannins [23,43], which tend to affect digestion processes through interaction with proteins and digestive enzymes [44]. The influence of dietary *T. molitor* inclusion seems to be both species-specific and dose-dependent. Although in some livestock the inclusion of carob pod meal has been previously reported to enhance body weight, feed conversion ratio and growing performance with no adverse effects on meat quality [28,45], other species exhibit no such changes in their growth under the effect of dietary carob pods inclusion [46,47]. In non-livestock animals such as rats, carob dietary fiber inclusion exhibits no statistically relevant effect on body weight [48]. Final individual larval weight and development time was strongly affected by the composition of the experimental substrates. Substrates with higher fat and energy content have been shown to increase larval weight [11], and in this study, a positive correlation was also observed. More amylaceous substrates have been shown to promote growth in TM larvae [49] and according to the documented starch contents of wheat bran [50] and carob pods [51], the starch content of the experimental substrates was decreased with the increase of carob content. However, despite the differences in the nutrient composition of the experimental substrates, only the 100% substrate resulted in a significantly lower final larval weight. Regarding survival, it has been found that high protein substrates promote larval survival [52], however, in this study, the survival of the larvae did not show a clear correlation with the protein content or the nutrient composition in general.

The nutrient composition of the experimental larvae of *T. molitor* was assessed in the present study, given that diet can have a significant effect on the proximate composition of the larvae [53,54,55]. Larval dry matter was not affected by the different substrates and their respective nutrient composition. Protein content ranged between 58.9% and 64.8% (Kp = 6.25 or 44.8–49.4% with Kp = 4.76), with larvae reared on 75% and 100% carob substrates displaying significantly higher protein content compared to the control group (0%). In this study, larvae protein content was slightly higher than the documented values for *T. molitor* larvae (47.2–60.3% [54]) which is favorable, since insect meals are extensively studied due to their rich protein content. Generally, insect larvae protein content has been shown to positively correlate with the substrate’s protein and fat contents [55,56]. However, herein such a correlation was not observed; *T. molitor* larvae were able to utilize efficiently the poorer substrates in these nutrients (75% and 100%) and convert them into a substantial amount of animal protein. Regarding fat content, no influence was observed in response to the different substrates, despite the almost two-fold difference in the fat content among the control and the 100% carob substrate. However, a moderate positive correlation was observed between the larvae and substrate’s fat and energy contents. Generally, an increase in the fat content of *T. molitor* larvae fed substrates rich in fat has been observed [11,53,56]. In this study, the energy content was higher in larvae fed 0% and 50% carob substrates compared to the 75% and 100% groups, and larval energy content appeared to be affected by the fat and energy content of the substrates. Chitin content was increased in the groups fed substrates with higher carob content levels (50, 75 and 100%). In this study, the nutrient composition of the larvae was strongly influenced by the final weight of the larvae, with larger insect larvae having higher fat content as well as lower protein and chitin contents. These results could be attributed to the lower carob content substrates, which are rich in fat and carbohydrates, thus promoting fat accumulation.

The data presented herein regarding lipid peroxidation and antioxidant enzymatic defenses show that the 50% milled carob pods content resulted in the lowest peroxidation levels, as suggested by TBARS levels. Thus, at this content level, oxidative damage was at its lowest compared to other substrates and the same was true for the activity levels of antioxidant enzymes, probably due to non-required antioxidant defense. Similarly, the concentration of TBARS decreases significantly in growing rabbits fed with carob pods. However, contrary to our results, the activities of glutathione peroxidase, glutathione S-transferase, catalase and superoxide dismutase may display an increase [28]. This antioxidant effect of carob is usually attributed to its total polyphenols, total flavonoids and condensed tannins content [57]. These molecules are the primal source of the antioxidant ability of this plant, by scavenging free radicals as hydroxyl radicals (OH^•^), which is the major cause of lipid peroxidation [58]. However, as observed in the present study by the results regarding carob’s antioxidant capacity, the >75% content decreased its antioxidant potential. The latter may be explained on the basis of possible saturation around the 75% carob content, formation of chemical complexes, and, consequently, precipitation, leading to lower values in the 100% carob content; it can also be attributed to the fact that some known antioxidants have been reported to have prooxidant behavior due to the presence of metal ions, the concentration of the given antioxidant in matrix environments and its redox potential [59,60,61]. To our knowledge, no literature exists about the effect of carob on the antioxidant enzymatic defense of non-stressed insects potentially destined for food consumption. However, several studies have been conducted in mammals under stress conditions such as EtOH-induced oxidative stress, CCl4 induced toxicity and streptozotocin-induced diabetes [27,62,63].

The induction of heat shock proteins (HSPs) and/or the activation of mitogen-activated protein kinases (MAPKs), both of which are evolutionarily conserved and present in all organisms ranging from invertebrates (e.g., [64,65]) to teleosts (e.g., [66]) and mammals (e.g., [67]), are considered to be among the adaptive cellular responses to miscellaneous stressors. Previous studies have demonstrated that changes in the dietary regime induce the gene and protein expression of HSPs in animals including *Pteromalus puparum* and *Drosophila melanogaster* [68,69,70,71]. Herein, the partial milled whole carob content in the feeding substrate favored the reduction of HSPs levels and p38 activation in reared *T. molitor*. Moreover, HSP70 and activation of p38 MAPK exhibited a similar pattern with regard to the increasing carob content level, which is in agreement with the involvement of p38 MAPK activation in other organisms, such as teleosts’ adaptations to thermal stress through the induction of HSP70 [66]. However, milled whole carob as a sole substrate induced both HSP90 and HSP60 expression, indicating a potential physiological activation of stress responses. Specifically, HSPs are known to be vital components in insects’ tolerance mechanisms to extreme environmental conditions and, as molecular chaperones, they are involved in the prevention of stress-mediated protein unfolding, subsequently ensuring the maintenance of cell homeostasis [72,73]. It is known that consumption of high amounts of condensed tannins, which are commonly present in carob pods [23], may actually lead to metabolic stress and thus may disrupt the animals’ welfare due to their anti-nutritional effects, resulting in reduced growth, protein digestibility and feed efficiency [74,75]. Nevertheless, different animals may exhibit variations in the tolerance to anti-nutritional factors [74]. It should be noted that insects are ancient organisms in evolutionary terms, and as such their adaptation to cope with such chemicals has been developed quite earlier during evolution compared to mammals [76]. The latter may explain the normal larval growth with no stress indications exhibited herein and the tolerance of *T. molitor* to higher content levels of milled whole carob compared to other animals. Certainly, further investigations are needed to shed light in such adaptations, and these should study both short-term and long-term effects of rearing with carob as novel substrate.

It is well established nowadays that excess cellular levels of ROS cause damage to proteins, nucleic acids, lipids, membranes and organelles, which can lead to activation of cell death processes such as apoptosis [77]. The linkage between oxidative stress and apoptosis is also mirrored in the results herein since Bax/Bcl-2 ratio and lipid peroxidation seemed to decrease in parallel. To our knowledge, although there is no information linking carob and cell death in animals, research on carob’s effect on the apoptotic pathway has been extensively studied in cancer cells and cancer cell lines. These studies have shown that carob induces apoptosis in cancer cells and inhibits their proliferation (e.g., [78,79,80]). The underlying mechanism is attributed to the inhibition of the activation of the AKT pathway, which in turn leads to the activation of the apoptotic pathway [79]. In the present study, a concentration-dependent beneficial effect on *T. molitor* larvae was shown. However, the extent of supplementation with antioxidants such as carob is still debatable since more data and validation are required.

## 5. Conclusions

The study herein explored for the first time the utilization of milled whole carob pods as an alternative and potentially beneficial substrate for the rearing of yellow mealworms. The results showed that carob content up to 75% did not significantly differentiate the larvae weight, developmental time and total dry matter. The total phenolic content of the larvae and their antioxidant activity exhibited a significant increase at 75% carob content. Although the antioxidant enzymes’ activity was decreased at both 25 and 50% carob content levels, higher content levels (75 and 100%) resulted in no significant changes compared to the control. Carob pods led to decreased apoptotic indicators and the low expression of most stress-related proteins when compared to the control. The present findings demonstrate that carob pods and their antioxidant properties exert beneficial effects on *T. molitor*’s rearing and nutritional status, although 100% carob content may adversely impact the larvae due to the high amounts of carob tannins, which act as anti-nutritional factors. Moreover, the 100% substrate probably lacked wheat bran, and thus nutritional factors necessary for animal development. Although the up to 75% carob content was beneficial, the consumption by insects of high amounts of condensed carob tannins at full substitution of the wheat bran may have induced their metabolic stress mechanisms, thus exerting a strong anti-nutritional effect.

## Figures and Tables

**Table 1:** Proximate composition of the experimental substrates used for the rearing of *Tenebrio molitor* fed a control wheat bran substrate (0%) and substrates incorporating 25, 50, 75 or 100% carob meal.

Carob Content	Dry Matter (%)	Protein (%)	Fat (%)	Ash (%)	Energy (MJ/kg)
0%	89.4 ± 1.5	16.6 ± 0.2	4.6 ± 0.2	5.1 ± 0.6	18.4 ± 0.1
25%	88.6 ± 0.4	15.5 ± 0.5	4.9 ± 0.4	5.1 ± 0.7	18.4 ± 0.2
50%	89.1 ± 0.5	17.2 ± 0.5	3.8 ± 0.2	6.2 ± 0.8	18.0 ± 0.2
75%	89.5 ± 0.5	15.1 ± 0.8	3.2 ± 0.4	6.9 ± 1.2	17.4 ± 0.2
100%	90.2 ± 0.3	14.2 ± 1.9	2.8 ± 0.4	9.0 ± 0.7	16.7 ± 0.2

Mean ± SD (*n* = 3), in dry mater basis.

**Table 2:** Proximate composition of *Tenebrio molitor* larvae fed a control wheat bran substrate (0%) or substrates incorporating 25, 50, 75 or 100% carob meal.

Carob Content	Dry Matter (%)	Protein Nx 6.25 (%)	Protein Nx4.76 (%)	Fat (%)	Ash (%)	Energy (MJ/kg)	Chitin (%)
0%	36.0 ± 1.5	58.9 ± 0.8 ^b^	44.8 ± 0.6 ^b^	23.8 ± 0.4 ^a^	5.9 ± 0.2	25.3 ± 0.1 ^a^	6.4 ± 0.0 ^b^
25%	36.2 ± 1.2	61.2 ± 1.7 ^a,b^	46.6 ± 1.3 ^a,b^	23.6 ± 1.1 ^a,b^	4.9 ± 0.9	25.1 ± 0.1 ^a,b^	7.1 ± 0.7 ^b^
50%	34.1 ± 1.4	62.1 ± 0.8 ^a,b^	47.3 ± 0.6 ^a,b^	20.7 ± 1.1 ^b^	4.6 ± 0.2	25.6 ± 0.6 ^a^	8.4 ± 0.2 ^a^
75%	35.2 ± 0.2	64.5 ± 3.2 ^a^	49.1 ± 2.4 ^a^	23.0 ± 1.7 ^a,b^	4.6 ± 0.7	24.2 ± 0.4 ^b^	8.4 ± 0.4 ^a^
100%	35.4 ± 2.0	64.8 ± 1.3 ^a^	49.4 ± 1.0 ^a^	21.9 ± 0.9 ^a,b^	4.2 ± 1.2	24.2 ± 0.3 ^b^	8.2 ± 0.2 ^a^

Mean ± SD (*n* = 3), on dry mater basis; Different letters in each column indicate statistically significant difference *p* < 0.05.

## Data Availability

All of the data is contained within the article and Supplementary Materials.

## Supplementary Materials

The following supporting information can be downloaded at: https://www.mdpi.com/article/10.3390/antiox11091840/s1, Figure S1: Complete original immunoblots shown in Figure 6; Figure S2: Complete original immunoblots shown in Figure 7; Figure S3: Complete original immunoblots shown in Figure 8; Figure S4: Complete original immunoblots shown in Figure 6, Figure 7 and Figure 8.

## References

[1] FAO (2022). The State of World Fisheries and Aquaculture. Towards Blue Transformation.

[2] Salter A.M., Lopez-Viso C. (2021). Role of novel protein sources in sustainably meeting future global requirements. Proc. Nutr. Soc..

[3] Berggren Å., Jansson A., Low M. (2019). Approaching ecological sustainability in the emerging insects-as-food industry. Trends Ecol. Evol..

[4] van Huis A., van Itterbeeck J., Klunder H., Mertens E., Halloran A., Muir G., Vantomme P. (2013). Edible Insects: Future Prospects for Food and Feed Security.

[5] van Huis A. (2022). Edible insects: Non-food and non-feed industrial applications. J. Insects Food Feed..

[6] Finke M.D., Oonincx D.G.A.B., Morales-Ramos J.A.R., Rojas M.G., Shapirollan D.I. (2014). Insects as food for insectivores. Mass Production of Beneficial Organisms: Invertebrates and Entomopathogens.

[7] Bessa L.W., Pieterse E., Sigge G., Hoffman L.C. (2020). Insects as human food; from farm to fork. J. Sci. Food Agric..

[8] Van Huis A., Oonincx D.G.A.B. (2017). The environmental sustainability of insects as food and feed. A review. Agron. Sustain. Dev..

[9] Oonincx D.G.A.B., De Boer I.J.M. (2012). Environmental impact of the production of mealworms as a protein source for humans—A life cycle assessment. PLoS ONE.

[10] Stahel W.R. (2016). The circular economy. Nature.

[11] Andreadis S.S., Panteli N., Mastoraki M., Rizou E., Stefanou V., Tzentilasvili S., Sarrou E., Chatzifotis S., Krigas N., Antonopoulou E. (2022). Towards functional insect feeds: Agri-food by-products enriched with post-distillation residues of medicinal aromatic plants in *Tenebrio molitor* (Coleoptera: *Tenebrionidae*) breeding. Antioxidants.

[12] Cappellozza S., Leonardi M.G., Savoldelli S., Carminati D., Rizzolo A., Cortellino G., Terova G., Moretto E., Badaile A., Concheri G. (2019). A first attempt to produce proteins from insects by means of a circular economy. Animals.

[13] Bava L., Jucker C., Gislon G., Lupi D., Savoldelli S., Zucali M., Colombini S. (2019). Rearing of *Hermetia illucens* on different organic by-products: Influence on growth, waste reduction, and environmental impact. Animals.

[14] Mastoraki M., Ferrándiz P.M., Vardali S.C., Kontodimas D.C., Kotzamanis Y.P., Gasco L., Chatzifotis S., Antonopoulou E. (2020). A comparative study on the effect of fish meal substitution with three different insect meals on growth, body composition and metabolism of European sea bass (*Dicentrarchus labrax* L.). Aquaculture.

[15] Mastoraki M., Katsika L., Enes P., Guerreiro I., Kotzamanis Y.P., Gasco L., Chatzifotis S., Antonopoulou E. (2022). Insect meals in feeds for juvenile gilthead seabream (*Sparus aurata*): Effects on growth, blood chemistry, hepatic metabolic enzymes, body composition and nutrient utilization. Aquaculture.

[16] Panteli N., Mastoraki M., Lazarina M., Chatzifotis S., Mente E., Kormas K.A., Antonopoulou E. (2021). Configuration of gut microbiota structure and potential functionality in two teleosts under the influence of dietary insect meals. Microorganisms.

[17] Antonopoulou E., Nikouli E., Piccolo G., Gasco L., Gai F., Chatzifotis S., Mente E., Kormas K.A. (2019). Reshaping gut bacterial communities after dietary *Tenebrio molitor* larvae meal supplementation in three fish species. Aquaculture.

[18] Mente E., Bousdras T., Feidantsis K., Panteli N., Mastoraki M., Kormas K.A., Chatzifotis S., Piccolo G., Gasco L., Gai F. (2022). *Tenebrio molitor* larvae meal inclusion affects hepatic proteome and apoptosis and/or autophagy of three farmed fish species. Scient. Rep..

[19] Zohary D. (2002). Domestication of the carob (*Ceratonia siliqua* L.). Isr. J. Plant Sci..

[20] Correia P.J., Gama F., Pestana M., Martins-Loução M.A. (2010). Tolerance of young (*Ceratonia siliqua* L.) carob rootstock to NaCl. Agric. Water Manag..

[21] Batlle I. (1997). Carob Tree: Ceratonia siliqua L. Promoting the Conservation and Use of Underutilized and Neglected Crops.

[22] Manso T., Nunes C., Raposo S., Lima-Costa M.E. (2010). Carob pulp as raw material for production of the biocontrol agent *P. agglomerans* PBC-1. J. Ind. Microbiol. Biotechnol..

[23] Avallone R., Plessi M., Baraldi M., Monzani A. (1997). Determination of chemical composition of carob (*Ceratonia siliqua*): Protein, fat, carbohydrates, and tannins. J. Food Compos. Anal..

[24] Ayaz F.A., Torun H., Ayaz S., Correia P.J., Alaiz M., Sanz C., Grúz J., Strnad M. (2007). Determination of chemical composition of anatolian carob pod (*Ceratonia siliqua* L.): Sugars, amino and organic acids, minerals and phenolic compounds. J. Food Qual..

[25] Youssef M.K.E., El-Manfaloty M.M., Ali H.M. (2013). Assessment of proximate chemical composition, nutritional status, fatty acid composition and phenolic compounds of carob (*Ceratonia siliqua* L.). Food Public Health.

[26] Scalbert A., Manach C., Morand C., Rémésy C., Jiménez L. (2005). Dietary polyphenols and the prevention of diseases. Crit. Rev. Food Sci. Nutr..

[27] Rtibi K., Jabri M.A., Selmi S., Souli A., Sebai H., El-Benna J., Amri M., Marzouki L. (2015). Gastroprotective effect of carob (*Ceratonia siliqua* L.) against ethanol-induced oxidative stress in rat. BMC Complement. Med. Ther..

[28] Abu Hafsa S.H., Ibrahim S.A., Hassan A.A. (2017). Carob pods (*Ceratonia siliqua* L.) improve growth performance, antioxidant status and caecal characteristics in growing rabbits. J. Anim. Physiol. Anim. Nutr..

[29] AOAC (Association of Official Analytical Chemists) (1990). Official Methods of Analysis.

[30] Folch J., Lees M., Sloane Stanley G.H. (1957). A simple method for the isolation and purification of total lipids from animal tissues. J. Biol. Chem..

[31] Janssen R.H., Vincken J.P., van den Broek L.A.M., Fogliano V., Lakemond C.M.M. (2017). Nitrogen-to-protein conversion factors for three edible insects: *Tenebrio molitor*, *Alphitobius diaperinus*, and *Hermetia illucens*. J. Agric. Food Chem..

[32] Finke M.D. (2007). Estimate of chitin in raw whole insects. Zoo Biol..

[33] Goering H.K., Van Soest P.J. (1970). Forage fiber analysis (apparatus, reagents, procedures, and some applications). Agriculture Handbook United States Department of Agriculture.

[34] Marono S., Piccolo G., Loponte R., Di Meo C., Attia Y.A., Nizza A., Bovera F. (2015). In vitro crude protein digestibility of *Tenebrio molitor* and *Hermetia illucens* insect meals and its correlation with chemical composition traits. Ital. J. Anim. Sci..

[35] Frühbauerová M., Červenka L., Hájek T., Pouzar M., Palarčík J. (2022). Bioaccessibility of phenolics from carob (*Ceratonia siliqua* L.) pod powder prepared by cryogenic and vibratory grinding. Food Chem..

[36] Kyriacou M.C., Emmanouilidou M.G., Soteriou G.A. (2016). Asynchronous ripening behavior of cactus pear (*Opuntia ficus-indica*) cultivars with respect to physicochemical and physiological attributes. Food Chem..

[37] Risaliti L., Kehagia A., Daoultzi E., Lazari D., Bergonzi M.C., Vergkizi-Nikolakaki S., Hadjipavlou-Litina D., Bilia A.R. (2019). Liposomes loaded with *Salvia triloba* and *Rosmarinus officinalis* essential oils: In vitro assessment of antioxidant, antiinflammatory and antibacterial activities. J. Drug Deliv. Sci. Technol..

[38] Salach J.I., Fleischer S., Packer L. (1978). Preparation of monoamine oxidase from beef liver mitochondria. Methods Enzymol..

[39] Buege J.A., Aust S.D., Fleischer S., Packer L. (1978). Microsomal lipid peroxidation. Methods Enzymol..

[40] Carlberg I., Mannervik B., Meister A. (1985). Glutathione reductase. Methods Enzymol..

[41] Cohen G., Dembiec D., Marcus J. (1970). Measurement of catalase activity in tissue extracts. Anal. Biochem..

[42] Paoletti F., Mocali A. (1990). Determination of superoxide dismutase activity by purely chemical system based on NAD(P)H oxidation. Methods Enzymol..

[43] Kumazawa S., Taniguchi M., Suzuki Y., Shimura M., Kwon M.-S., Nakayama T. (2002). Antioxidant activity of polyphenols in carob pods. J. Agric. Food Chem..

[44] Silanikove N., Landau S., Or D., Kababya D., Bruckental I., Nitsan Z. (2006). Analytical approach and effects of condensed tannins in carob pods (*Ceratonia siliqua*) on feed intake, digestive and metabolic responses of kids. Livest. Sci..

[45] Kotrotsios N., Christaki E., Bonos E., Florou-Paneri P. (2012). Dietary carob pods on growth performance and meat quality of fattening pigs. Asian-Australas. J. Anim. Sci..

[46] Priolo A., Lanza M., Biondi L., Pappalardo P., Young O. (1998). Effect of partially replacing dietary barley with 20% carob pulp on post-weaning growth, and carcass and meat characteristics of Comisana lambs. Meat Sci..

[47] Sahle M., Coleou J., Haas C. (1992). Carob pod (*Ceratonia siliqua*) meal in geese diets. Br. Poult. Sci..

[48] Burget L., Caton S., Bai Y., Spangler L., Gruendel S., Koebnick C., Bidlingmaier M. (2007). Longterm effects of carob pulp preparations in insoluble fiber on metabolism, body weight and leptin levels in rats. Exp. Clin. Endocrinol. Diabetes.

[49] Rumbos C.I., Karapanagiotidis I.T., Mente E., Psofakis P., Athanassiou C.G. (2020). Evaluation of various commodities for the development of the yellow mealworm, *Tenebrio molitor*. Sci. Rep..

[50] Heuzé V., Tran G., Baumont R., Noblet J., Renaudeau D., Lessire M., Lebas F. Wheat Bran. Feedipedia, a Programme by INRAE, CIRAD, AFZ and FAO. https://www.feedipedia.org/node/726.

[51] Heuzé V., Tran G., Sauvant D., Lessire M., Lebas F. Carob (*Ceratonia siliqua*). Feedipedia, a Programme by INRAE, CIRAD, AFZ and FAO. https://www.feedipedia.org/node/320.

[52] Oonincx D.G.A.B., Van Broekhoven S., Van Huis A., van Loon J.J.A. (2015). Feed conversion, survival and development, and composition of four insect species on diets composed of food by-products. PLoS ONE.

[53] van Broekhoven S., Oonincx D.G.A.B., van Huis A., van Loon J.J.A. (2015). Growth performance and feed conversion efficiency of three edible mealworm species (Coleoptera: *Tenebrionidae*) on diets composed of organic by-products. J. Insect Physiol..

[54] Dreassi E., Cito A., Zanfini A., Materozzi L., Botta M., Francardi V. (2017). Dietary fatty acids influence the growth and fatty acid composition of the yellow mealworm *Tenebrio molitor* (Coleoptera: *Tenebrionidae*). Lipids.

[55] Kröncke N., Benning R. (2022). Self-selection of feeding substrates by *Tenebrio molitor* larvae of different ages to determine optimal macronutrient intake and the influence on larval growth and protein content. Insects.

[56] Tran G., Gnaedinger C., Mélin C. Mealworm (*Tenebrio molitor*). Feedipedia, a Programme by INRAE, CIRAD, AFZ and FAO. https://feedipedia.org/node/16401.

[57] Hichem S., Abdelaziz S., Latifa C., Kais R., Mohamed A., Jamel E.-B., Mohsen S. (2013). In vitro and in vivo antioxidant properties of Tunisian carob (*Ceratonia siliqua* L.). J. Med. Plant Res..

[58] Kogiannou D.A., Kalogeropoulos N., Kefalas P., Polissiou M.G., Kaliora A.C. (2013). Herbal infusions; their phenolic profile, antioxidant and anti-inflammatory effects in HT29 and PC3 cells. Food Chem. Toxicol..

[59] Ionescu J.G., Novotny J., Stejskal V., Laetsch A., Blaurock-Busch E., Eisenmann-Klein M. (2006). Increased levels of transition metals in breast cancer tissue (Erratum in: Neuro Endocrinol Lett. 2007 Oct; 28(5): iii; PMID: 16804515). Neuro Endocrinol. Lett..

[60] Ionescu J.G., Merk M., Dowes F. Clinical application of redox potential testing in the blood. Proceedings of the 33rd Annual Meeting of the American Academy Environmental Medicine.

[61] González M.J., Miranda-Massari J.R., Mora E.M., Guzmán A., Riordan N.H., Riordan H.D., Casciari J.J., Jackson J.A., Román-Franco A. (2005). Orthomolecular oncology review: Ascorbic acid and cancer 25 years later. Integr. Cancer Ther..

[62] El-Haskoury R., Al-Waili N., Kamoun Z., Makni M., Al-Waili H., Lyoussi B. (2018). Antioxidant activity and protective effect of carob honey in CCl4-induced kidney and liver injury. Arch. Med. Res..

[63] El-Haskoury R., Al-Waili N., El-Hilaly J., Al-Waili W., Lyoussi B. (2019). Antioxidant, hypoglycemic, and hepatoprotective effect of aqueous and ethyl acetate extract of carob honey in streptozotocin-induced diabetic rats. Vet. World.

[64] Chen J., Xie C., Tian L., Hong L., Wu X., Han J. (2010). Participation of the p38 pathway in *Drosophila* host defense against pathogenic bacteria and fungi. Proc. Nat. Acad. Sci. USA.

[65] Demertzioglou M., Antonopoulou E., Voutsa D., Kozari A., Moustaka-Gouni M., Michaloudi E. (2021). MAPKs and HSPs’ activation of a natural *Daphnia magna* population in a man-perturbed lake: Implications of ecological significance. Water.

[66] Feidantsis K., Poertner H.O., Markou T., Lazou A., Michaelidis B. (2012). Involvement of p38 MAPK in the induction of Hsp70 during acute thermal stress in red blood cells of the gilthead sea bream, *Sparus aurata*. J. Exp. Zool. A Ecol. Genet. Physiol..

[67] Xie F., Zhan R., Yan L.-C., Gong J.-B., Zhao Y., Ma J., Qian L.-J. (2016). Diet-induced elevation of circulating HSP70 may trigger cell adhesion and promote the development of atherosclerosis in rats. Cell Stress Chaperones.

[68] Wang H., Li K., Zhu J.Y., Fang Q., Ye G.Y., Wang H., Li K., Zhu J.Y. (2012). Cloning and expression pattern of heat shock protein genes from the endoparasitoid wasp *Pteromalus puparum* in response to environmental stresses. Arch. Insect Biochem. Physiol..

[69] Antonopoulou E., Kentepozidou E., Feidantsis K., Roufidou C., Despoti S., Chatzifotis S. (2013). Starvation and re-feeding affect Hsp expression, MAPK activation and antioxidant enzymes activity of European sea bass (*Dicentrarchus labrax*). Comp. Biochem. Physiol. A.

[70] Feidantsis K., Kaitetzidou E., Mavrogiannis N., Michaelidis B., Kotzamanis Y., Antonopoulou E. (2014). Effect of taurine-enriched diets on the Hsp expression, MAPK activation and the antioxidant defence of the European sea bass (*Dicentrarchus labrax*). Aquacult. Nutr..

[71] Pandey A., Vimal D., Chandra S., Saini S., Narayan G., Kar Chowdhuri D. (2014). Long-term dietary exposure to low concentration of dichloroacetic acid promoted longevity and attenuated cellular and functional declines in aged *Drosophila melanogaster*. Age.

[72] Becker J., Craig E.A. (1994). Heat-shock proteins as molecular chaperones. Eur. J. Biochem..

[73] King A.M., MacRae T.H. (2015). Insect heat shock proteins during stress and diapause. Ann. Rev. Entomol..

[74] Gobindram M.N.N.E., Bognanno M., Luciano G., Lanza M., Biondi L. (2015). Carob pulp inclusion in lamb diets: Effect on intake, performance, feeding behaviour and blood metabolites. Anim. Prod. Sci..

[75] Gemede H.F., Ratta N. (2014). Antinutritional factors in plant foods: Potential health benefits and adverse effects. Int. J. Food Sci. Nutr..

[76] Bernays E.A. (1978). Tannins: An alternative viewpoint. Entomol. Exp. Appl..

[77] Redza-Dutordoir M., Averill-Bates D.A. (2016). Activation of apoptosis signaling pathways by reactive oxygen species. Biochim. Biophys. Acta Mol. Cell. Res..

[78] Corsi L., Avallone R., Cosenza F., Farina F., Baraldi C., Baraldi M. (2002). Antiproliferative effects of Ceratonia siliqua L. on mouse hepatocellular carcinoma cell line. Fitoterapia.

[79] Gregoriou G., Neophytou C.M., Vasincu A., Gregoriou Y., Hadjipakkou H., Pinakoulaki E., Christodoulou M.C., Ioannou G.D., Stavrou I.J., Christou A. (2021). Anti-cancer activity and phenolic content of extracts derived from Cypriot carob (*Ceratonia siliqua* L.) pods using different solvents. Molecules.

[80] Pouresmaeil V., Haghighi S., Raeisalsadati A.S., Neamati A., Homayouni-Tabrizi M. (2021). The anti-breast cancer effects of green-synthesized zinc oxide nanoparticles using carob extracts. Anticancer Agents Med. Chem..

