# Supplementary material for: Carob (Ceratonia siliqua) as Functional Feed Is Beneficial in Yellow Mealworm (Tenebrio molitor) Rearing: Evidence from Growth, Antioxidant Status and Cellular Responses
Source: Antioxidants (Basel). 2022 Sep 19;11(9):1840. doi: 10.3390/antiox11091840 (PMC9495811; doi:10.3390/antiox11091840)

**Figure S1:** Complete original immunoblots shown in Figure 6.

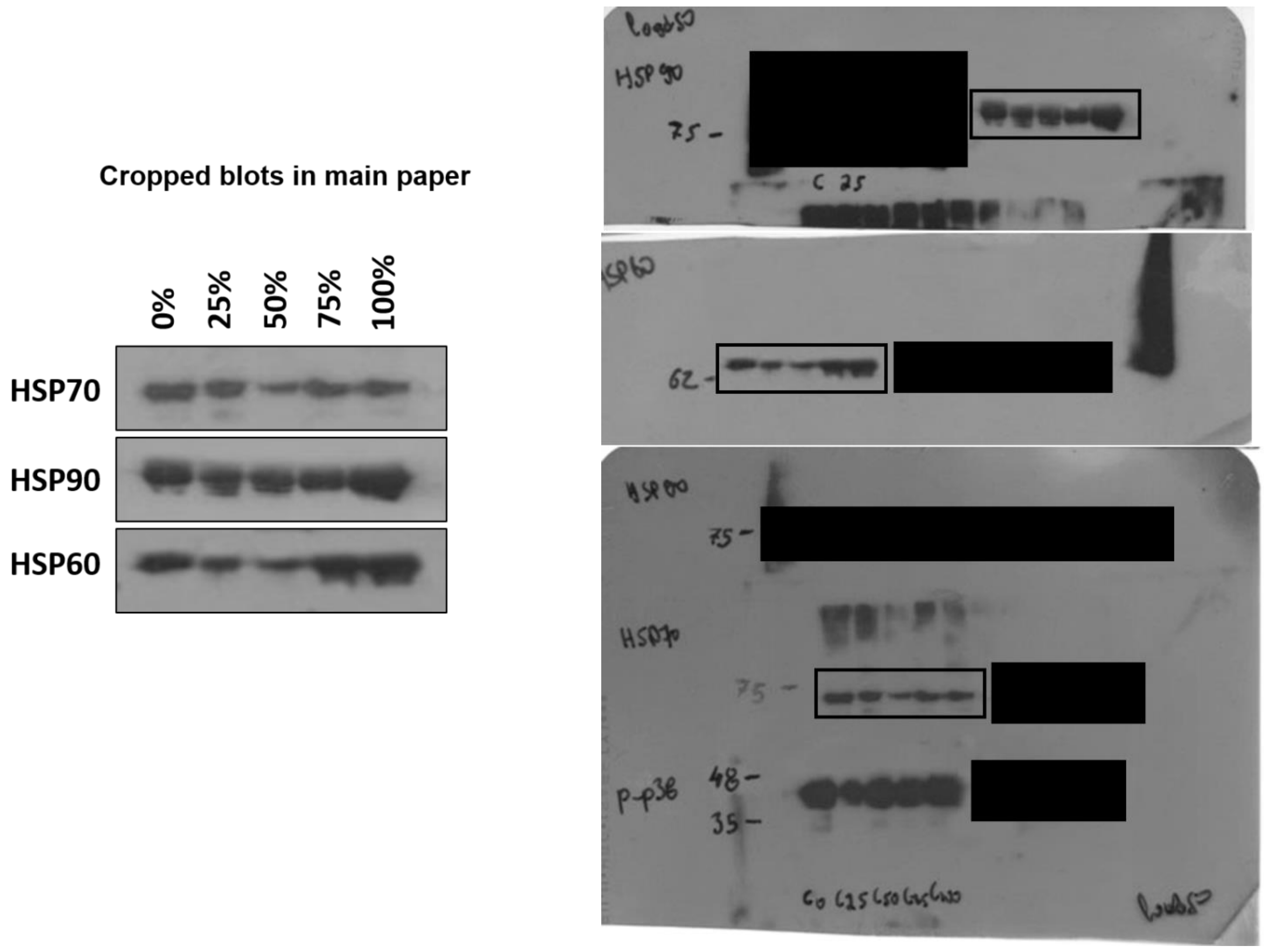

**Figure S2:** Complete original immunoblots shown in Figure 7.

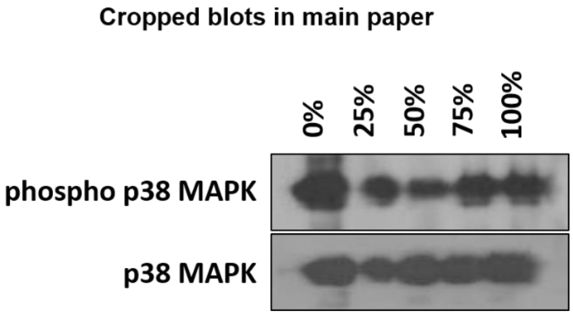


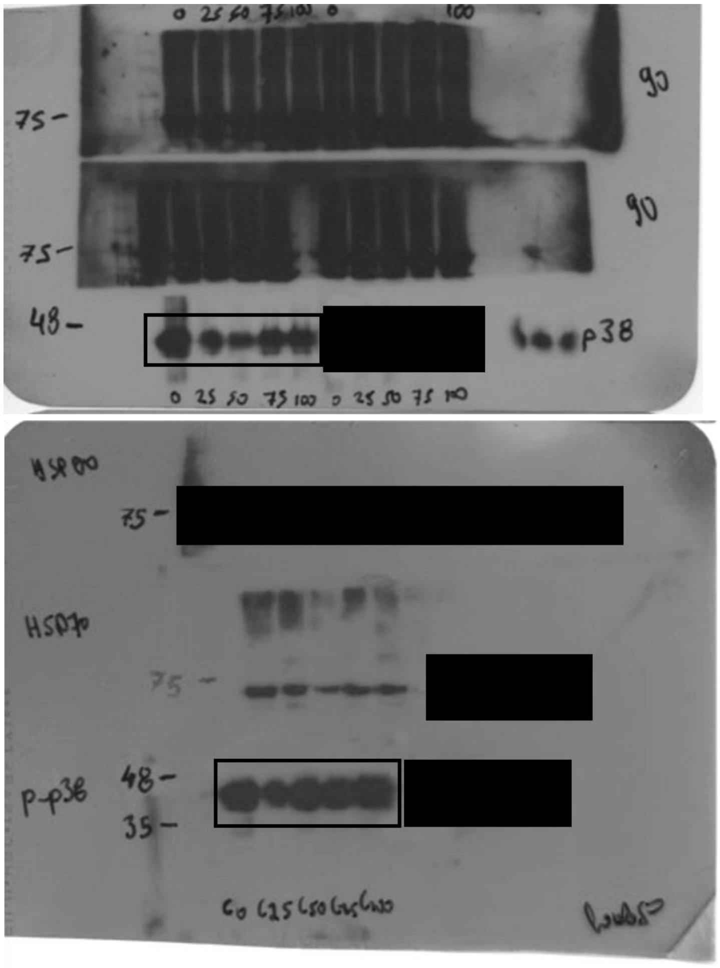

**Figure S3:** Complete original immunoblots shown in Figure 8.

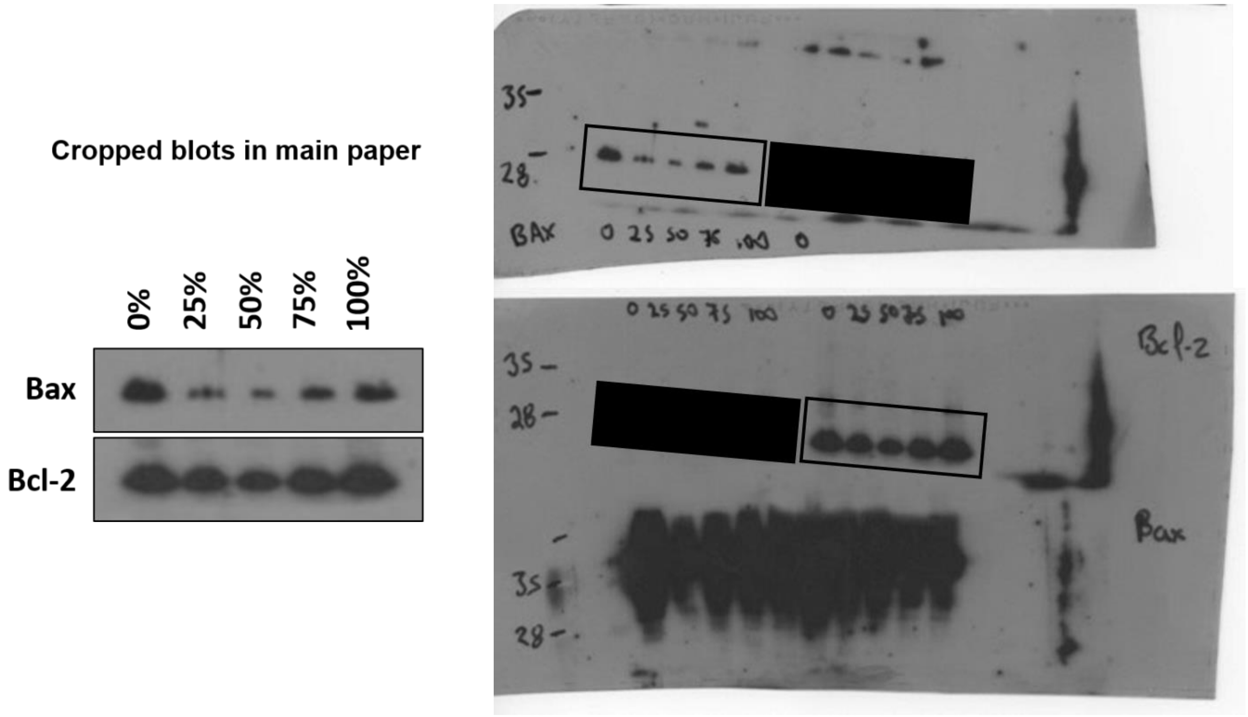

**Figure S4:** Complete original immunoblots shown in Figure 6, 7 and 8.

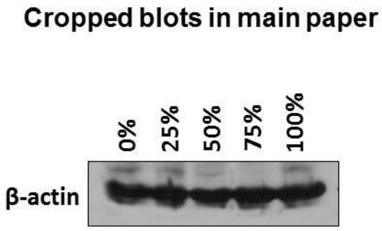


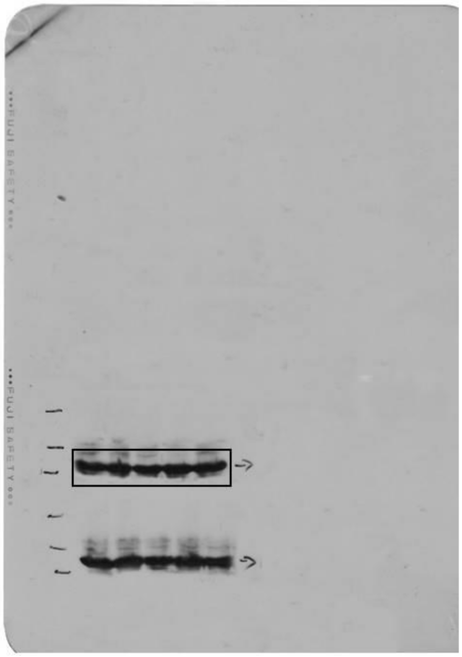

Supplement: Supplementary file 1 [file antioxidants-11-01840-s001.zip › antioxidants-1867533-supplementary.pdf]
